# Agave Bagasse as an Eco-Friendly Template for the Microwave-Assisted Synthesis of C@TiO_2_ Photoelectrodes

**DOI:** 10.3390/molecules31132399

**Published:** 2026-07-07

**Authors:** Patricia M. Olmos-Moya, Esmeralda Vences-Alvarez, Juan Matos, Marisol Aguilar, Sergio Velazquez-Martinez, Carlos Pineda-Arellano, Angel G. Rodríguez, Rene Rangel-Mendez, Luis F. Chazaro-Ruiz

**Affiliations:** 1 División de Ciencias Ambientales, Instituto Potosino de Investigación Científica y Tecnológica A.C., Camino a la Presa San José 2055, Col. Lomas 4a Sección, San Luis Potosí 78216, San Luís Potosí, Mexico; 2 Science and Engineering Division, University of Guanajuato, Lomas del Bosque #103, Lomas del Campestre, León 37150, Guanajuato, Mexico; 3 Grupo de Materiales Sostenibles Para la Circularidad (SMAC), Instituto Iberoamericano de Desarrollo Sostenible (IIDS), Facultad de Arquitectura, Construcción y Medio Ambiente, Universidad Autónoma de Chile, Temuco 4780000, Araucanía, Chile; 4 Centro de Investigaciones en Óptica, A.C., Unidad Aguascalientes, Prolongación Constitución 607, Fraccionamiento Reserva Loma Bonita, Aguascalientes 20200, Aguascalientes, Mexicocapia@cio.mx (C.P.-A.); 5 CONACYT-Centro de Investigación en Óptica, A.C. Prolongación Constitución 607, Fraccionamiento Reserva Loma Bonita, C. P. Aguascalientes 20200, Aguascalientes, Mexico; 6 Coordinación Para la Innovación y la Aplicación de la Ciencia y la Tecnología (CIACYT), Universidad Autónoma de San Luis Potosí (UASLP), Av. Sierra Leona 550, Lomas 2a Sección, San Luis Potosí 78216, San Luís Potosí, Mexico; angel.rodriguez@uaslp.mx

**Keywords:** agave bagasse waste, microwave-assisted synthesis, hybrid C@TiO_2_ composites, photoelectrodes, sensitized solar cells

## Abstract

This work reports, for the first time, the use of agave bagasse from “Tequila Weber Var” as an efficient and eco-friendly template for the microwave-assisted solvothermal synthesis of C@TiO_2_ photoelectrodes. The characterization of the C@TiO_2_ materials was performed using composition and elemental analysis, diffuse reflectance/UV-visible spectroscopy, N_2_ adsorption/desorption isotherms, scanning and transmission electron microscopy, energy-dispersive X-ray spectroscopy, X-ray diffraction patterns, cyclic voltammetry, impedance spectroscopy, and variations of the open-circuit potential in a conventional electrochemical cell. Three 1:1, 4:1, and 8:1 agave:Ti volume ratios were used to explore the influence of carbon content upon the optical and photoelectric properties of TiO_2_. The composite with a 1:1 ratio showed a charge transfer kinetic capacity of 0.86 C·cm^−2^·s^−1^ with the highest current density flow of 2.2 mA·cm^−2^, and the lowest optical band gap (E_bg_) value of 2.92 eV, boosting the optoelectronic behavior of TiO_2_. The photoanode composed of FTO/C@TiO_2_ with the hybrid material with a 1:1 ratio was preliminarily evaluated in a photovoltaic solar cell, showing a light-to-electricity conversion efficiency higher than the other two composites and up to 12.5 times higher than the photoanode only composed of neat TiO_2_. The present results contribute to the state-of-the-art of eco-friendly organic–inorganic thin film photoelectrodes for the sustainable synthesis of third-generation solar cells using bagasse-derived waste as an efficient carbon source for the synthesis of hybrid photoactive semiconductors.

## 1. Introduction

Solar energy is a renewable and environmentally friendly energy source that makes a viable and economical alternative to replace non-renewable energy sources. Photovoltaic cells are devices allowing the capture and conversion of solar-to-electrical energy [1,2]. Dye-sensitized solar cells (DSSCs) are part of the so-called third-generation cells that have received significant attention from the scientific community, but different challenges are still under study to achieve an efficient and low-cost development. DSSCs are complex systems [3] composed of the photoanode made of dye-sensitized photoactive semiconductors, the iodide/triiodide electrolyte, and the counter electrode. TiO_2_, ZnO, WO_3_, Nb_2_O_5_, and several heteroatom-doped composites [4] are the most used semiconductors for the manufacture of photoanodes. DSSCs are activated by the absorption of photons by the dye-sensitizer, while the semiconductor is responsible for the transfer of electrical charge from the activated state of the sensitizer to the external electrical circuit. TiO_2_ was originally [3] sensitized with dyes based on organometallic complexes, which have provided greater stability and efficiency than natural dyes extracted from plants. Nowadays, DSSCs are manufactured with photoanodes based on inorganic semiconductors and organic materials, that commonly are referred as hybrid organic–inorganic materials. TiO_2_ has been the most widely used semiconductor due to its high electrochemical stability. Accordingly, TiO_2_ is still an eminent issue in photocatalysis and photovoltaic fields; however, their scaling-up to real solar devices has been hindered due to its wide bandgap (ca. 3.2 eV and ca. 3.0 eV for anatase and rutile phases, respectively), whose excitation requires UV light, which only accounts for ca. 8% of the solar spectrum [5]. Furthermore, due to different variables including changes in the pH of the electrolyte, temperature and metallic impurities, the recombination of the photogenerated electron-hole pairs in TiO_2_ results in low conductivity [6].

Different approaches have been proposed to address the TiO_2_ limitations and improve its efficiency, including surface modification, doping with metals or non-metals, and decorating or functionalizing with carbon [7]. Hybrid inorganic semiconductors and natural organic compounds have been reported [8] with enhancements in the chemical resistance, photon absorption, and electrical conductivity of TiO_2_. The manufacture of hybrid photoelectrodes in the assembly of solar cells includes doped or sensitized semiconductors with polymers, carbon, and dyes derived from organic waste [9,10]. The main organic waste used includes peels, crab shells, bagasse, fish scales, coal, and paper [11]. In this context, Liu et al. [12] fabricated a counter photoelectrode based on a perovskite/carbon composite coupled to a solar cell. The carbon-based material was prepared by carbonization of soybean dregs. Dasari et al. [13] prepared a counter photoelectrode employing activated carbon obtained from the pyrolysis of coconut shell. Their photoelectrode was demonstrated to be a good harvester in the UV-visible light range and to have high stability for longer operation times. Wang et al. [14] reported the preparation of coal powder that increased the conductivity of photoelectrodes in assembled solar cells. Ma et al. [10] demonstrated that it is possible to prepare carbon-based counter photoelectrodes containing N, P, and S elements from carbonized fish biomass waste deposited on a FTO glass. Chen et al. [15] have reported the synthesis of photoelectrodes using carbon quantum dots (CQDs) from sugar cane bagasse as a carbon source with a photoelectric performance up to 13 times higher than bare TiO_2_.

In summary, carbon doping can substantially increase the photoactivity of TiO_2_, decreasing the energy band gap and permitting the absorption of light in the visible region [16]. C-doped TiO_2_ can also be able to trap and transport electrons, promoting a decrease in the charge recombination of photo-induced electron and hole pairs [17]. It is worth mentioning that graphitic carbon can act as an electrically conductive component, improving the diffusion of photogenerated electrons [18]. Matos et al. [19] prepared nanocrystalline C-doped TiO_2_ hybrid hollow spheres, using different carbon sources such as furfural, chitosan, or saccharose. In this work, the influence of carbon source upon the texture, crystalline framework, optical and photoelectrochemistry properties of TiO_2_ for its application as photoelectrodes was reported, highlighting a remarkable enhancement in the light harvesting efficiency of TiO_2_. Xu et al. [20] prepared a visible-light-driven C-modified TiO_2_ film on a conducting substrate and studied it as a photoanode, which exhibited enhanced electrochemical properties compared to unmodified TiO_2_. Rangel-Mendez et al. [21] synthesized C-doped TiO_2_ hybrid materials, which showed a lower energy band gap than the bare TiO_2_. This effect contributed to an enhancement in the photoelectrochemical performance for the application of C-doped TiO_2_ hybrid materials as photoelectrodes. Our group has also reported [22] a TiO_2_ material sensitized with CQDs obtained from orange peel waste. The hybrid CQDs/TiO_2_ showed a lower band gap compared to TiO_2_, promoting a better photovoltaic performance when the materials are used as photoelectrodes. Other strategies have also been reported for functionalizing TiO_2_ to obtain carbon-doped semiconductors for use in photocatalysis processes, such as biochar-derived activated carbon [23] or from glucose pyrolysis with metal-organic frameworks [24].

The present work explores the use of acid hydrolysate bagasse from agave Tequilana Weber Var from the tequila industry in Mexico as a sustainable source of C, P and N heteroatoms for the microwave-assisted solvothermal synthesis of hybrid C@TiO_2_ composites. The influence of the volume ratio agave:Ti, the temperature, and the reaction time upon the morphological, textural, optoelectronic and photoelectrochemical properties of TiO_2_ were verified, and the best material was selected for the manufacture of thin film photoelectrodes.

## 2. Results and Discussion

### 2.1. Characterization of the Materials

#### 2.1.1. Elemental Analysis and Composition of Agave Bagasse

It is important to highlight that bio-sourced materials like bagasse can have different batch-to-batch compositions. The performance of solar cells can be remarkably affected as a result of the composition variability in different batches of agave bagasse. Thus, it is mandatory to perform a careful characterization of the biomass source to establish the correct ranges of work for organic solar cells as a function of the chemical components of biomass waste. Regarding agave bagasse as a source of carbon and other heteroatoms, it is important to mention that being an endemic species of Mexico, the production of the original Agave Tequilana Weber Var is carried out with precaution, ensuring that its characteristics prevail in all batches, as it is the raw material for the production of “Tequila” with high international quality standards. Based on this, during the development of this work, we used agave bagasse wastes from two different batches, labeled as sample (A) and sample (B). We took three samples from each batch, and several analyses were performed to verify the homogeneity of the biomass.

Table S1 (Supplementary Information, SI), shows that the agave bagasse acid hydrolysate (AHAB) precursor is mainly composed of C, H, and O, and lower amounts of N, and S derived from the primary components of the agave bagasse [25], including cellulose, hemicellulose, and raw proteins (Table S2, SI).

Comparisons between the standard deviation and the average values in Table S1 (SI) yield percentual coefficients of variation (CV’s) of ca. 1.3%, 7.4%, 9.4%, 14.8%, and 0.6% for C, N, H, S, and O wt.% content, respectively. These CV values showed statistically acceptable results because the measures showed low variation around the average value (≤10%). In addition, the values of compositional analysis shown in Table S2 (SI) are even better, with percentual CV values of 0.03–0.16%. Finally, as can be seen in Figure S1 and Table S3 (SI), other elements such as P, Ca, Si, Zn, Al, and Na were also detected from inductively coupled plasma-optical emission spectroscopy (ICP-OES). The analysis of powders from sample A and sample B of agave bagasse showed low variation relative to the average value, with CV values much lower than 10%. Thus, it can be concluded that the present biomass waste is characterized by a high homogeneity in the chemical elemental analysis, and accordingly, it is expected that the materials prepared from these two batches showed a high reproducibility in their physicochemical properties.

The AHAB precursor together with titanium (IV) isopropoxide were the reagents (Section 3.1) used for the “bottom-up” solvothermal synthesis of C-containing TiO_2_ materials (Section 3.2), here denoted as C@TiO_2_ composites. It has been reported that solvothermal degradation of biomass-derived wastes is characterized by a series of reactions including condensation and amidation [26], aldol condensation [27], Schiff base [28], and self-polymerization [29]. In this work, three C@TiO_2_ composites with agave:Ti volume ratios of 1:1, 4:1, and 8:1 were reproducibly synthesized by a microwave-assisted solvothermal process, controlling temperature, pressure, and microwave power as the main reaction parameters. The formation of the composites by solvothermal synthesis has been reported to take place by a coalescence mechanism [30,31] involving both the condensation of carbon atoms from the thermal degradation of the biomass wastes and the hydrolysis of titanium alkoxide to yield Ti(OH)_4_ that is progressively dehydroxylated to TiO_2_, forming a hybrid composite here denoted as C@TiO_2_. As reported by our group [19,21], these materials are characterized by the embedding of other elements, that for the present work, besides carbon, phosphorus and nitrogen (Tables S1 and S3, SI) are also to be embedded.

#### 2.1.2. Optical Properties of C@TiO_2_ Composites

**Figure 1:**
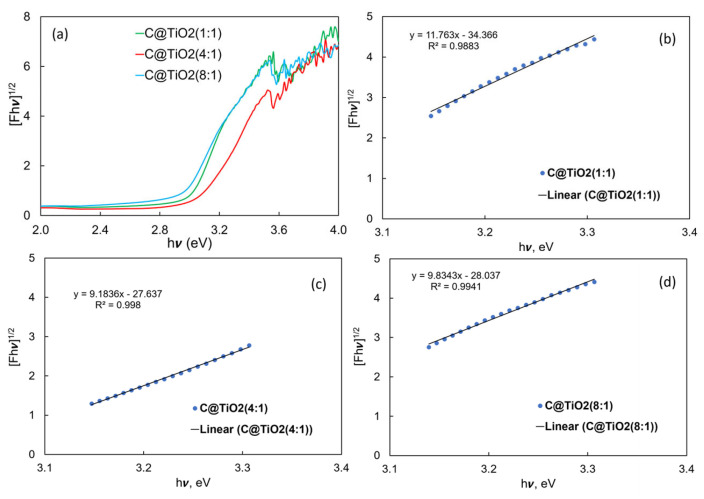
(**a**): Tauc plot of the composites in terms of the reflectance (R) as a function of photon energy. (**b**–**d**) Linear regressions of the Tauc plot in the energy range 3.13–3.30 eV.

The influence of the amount of AHAB as a carbon precursor upon the energy band gap (E_bg_) of C@TiO_2_ composites was evaluated by diffuse reflectance/UV-visible (DR/UV-Vis) spectroscopy. Figure 1a shows the Tauc plot of the reflectance (R) as a function of photon energy.

It can be seen that despite C@TiO_2_ (1:1) and C@TiO_2_ (8:1) having similar trends from 3.2–4.0 eV, it is clear that below 3.2 eV, the latter seems to have a lower wavelength shift. This trend is reasonable because the higher the carbon content in C@TiO_2_ composites, the higher the absorption of photons [19]. In contrast, C@TiO_2_ (4:1) shows the lower wavelength shift. The energy band gaps of the composites were estimated from the linear regressions (Figure 1b–d) of Tauc plot data [32]. The regressions were adjusted to the energy range 3.13–3.30 eV because it showed the best linear fit of the Tauc function for the present UV-visible data. According to the Kubelka-Munk formalism, the E_bg_ values were estimated assuming an indirect transition because anatase is the major crystalline phase observed in C@TiO_2_ composites, as discussed below from X-ray diffraction (XRD) patterns. The E_bg_ values are listed in Table 1, as well as the red shift observed for the C@TiO_2_ composites.

It can be seen that all the C@TiO_2_ composites prepared from 1:1, 4:1, and 8:1 volume ratios of agave:Ti showed a reduction in the E_bg_ compared to TiO_2_-P25. In addition, the standard deviation obtained in the E_bg_ values (from a triplicate analysis) showed absolute errors between 0.3–1.7%, suggesting the microwave-assisted solvothermal synthesis is a highly reproducible methodology to prepare C@TiO_2_ composites. It is interesting to highlight that increasing the carbon content in the composite, E_bg_ decreases from 2.92 eV up to 2.85 eV for C@TiO_2_ (1:1) and C@TiO_2_ (8:1), respectively. This result agrees with higher photon absorption in both UV and Visible light due to a higher presence of carbon [19,33].

However, as noted above, the C@TiO_2_ (4:1) composite shows a higher energy band gap than that observed on the C@TiO_2_ (8:1) composite (3.01 eV against 2.85 eV). This is an anomalous result, and we do not have an explanation for it, but in any case, this sample is also characterized by a lower E_bg_ than that of TiO_2_-P25. In addition, as observed from Figure 1 and Table 1, all C@TiO_2_ composites promote an important red-shift from 411–435 nm compared to the commercial TiO_2_-P25 (385 nm), in agreement with an enhancement of light harvesting efficiency in the visible range of the solar spectrum [19].

The present composites were calcined at low temperature (350 °C), and accordingly, as discussed below, a representative carbon content is still present in the composites. Therefore, it can be suggested that the lower E_bg_ observed in the C@TiO_2_ composites can be attributed to a bathochromic shift in the spectrum of TiO_2_ due to doping with other heteroatoms besides carbon, such as nitrogen and phosphorus [34]. This suggestion agrees with the contents detected in the AHAB precursor (Tables S1 and S3, SI). The influence of these heteroatoms is discussed below from the SEM-EDS analysis.

#### 2.1.3. Texture and Porosimetry of C@TiO_2_ Composites

**Figure 2:**
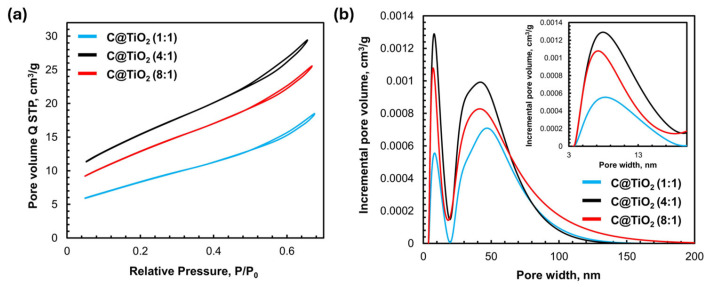
(**a**): N_2_ adsorption isotherms, and (**b**): Pore size distributions of C@TiO_2_ prepared from 1:1, 4:1 and 8:1 agave:Ti volume ratio.

In previous works [19,21], our group has reported that textural and porosimetry properties of C@TiO_2_ composites affect the light harvesting efficiency of potential photoanodes for their use as photovoltaic devices. The N_2_ adsorption/desorption isotherm and the pore size distribution (PSD) of the C@TiO_2_ composites are presented in Figure 2. It can be seen from Figure 2a that the N_2_ adsorption/desorption isotherms of the three C@TiO_2_ composites correspond to a type II isotherm characteristic of non-porous or meso/macroporous materials. In this study, the N_2_ isotherms of C@TiO_2_ materials showed a small H3-type hysteresis loop that agrees with the presence of mesopores, as can be seen in the PSD of Figure 2b.

The BET specific surface areas (S_BET_) of the C@TiO_2_ composite materials prepared from 1:1, 4:1 and 8:1 volume ratios were 31, 56 and 48 m^2^·g^−1^. These values were up to 10 times greater than those reported in previous work using microwave-assisted solvothermal synthesis [21], and the difference is attributed to the low calcination temperature (350 °C) used in the present work. It should be highlighted that the C@TiO_2_ composites presented a specific surface area like the commercial TiO2-P25 of ca. 50 m^2^·g^−1^ [21,22,33].

Contrary to the non-porous framework of TiO_2_-P25 (Table 1), the total pore volume of the present C@TiO_2_ composites was 0.029, 0.046 and 0.040 cm^3^·g^−1^ for the materials prepared from 1:1, 4:1 and 8:1 volume ratios, respectively, indicating the materials are mainly characterized by mesoporous materials with a fraction of large micropores, as can be seen from Figure 2b. C@TiO_2_ composites are characterized by an average pore size diameter in the range of large micropores estimated by the BJH (Barrett–Joyner–Halenda) equation.

However, the slight decrease in both S_BET_ and total pore volume observed in the C@TiO_2_ sample prepared from an 8:1 agave:Ti volume ratio (Table 1) compared to the sample prepared with a 4:1 volume ratio indicates that an excess of carbon content may act as a pore-blocking agent, also affecting the porous framework of TiO_2_ [35]. This inference is reasonable because the calcination temperature used in this work is only 350 °C, and it was confirmed by the high remaining C content observed for the samples from energy-dispersive X-ray spectroscopy (EDS) analysis discussed below. Representative values of surface area and total pore volume are beneficial in improving the photoelectrochemical kinetics, promoting high ion diffusion between the photoelectrode and the electrolyte solution [36], and improving the electrical conductivity. From the textural properties summarized in Table 1 can be concluded that the present materials are good enough to be analyzed as potential hybrid C@TiO_2_ photoelectrodes.

#### 2.1.4. SEM-EDS and HR-TEM Analysis of C@TiO_2_ Composites

**Figure 3:**
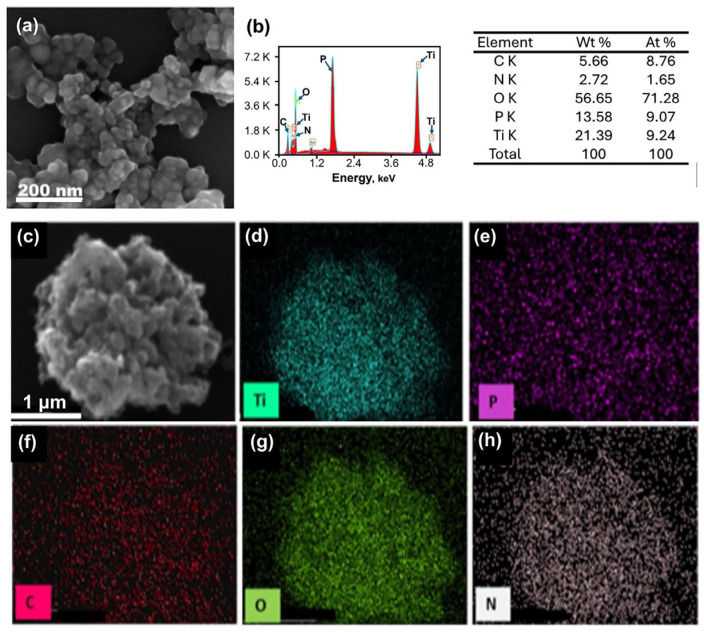
(**a**,**c**) SEM, (**b**) EDS, (**d**–**h**) SEM-EDS mapping of main elements detected on C@TiO_2_ (1:1) composite.

C@TiO_2_ (1:1) composite has been selected as a representative example to be analyzed by scanning electron microscopy (SEM), transmission electron microscopy (TEM), and energy-dispersive X-ray spectroscopy (EDS). The SEM micrographs in Figure 3a,c show that the C@TiO_2_ (1:1) composite is characterized by a rough surface and is mainly composed of clusters of spherical-like particles with a mean size of ca. 25.0 ± 0.2 nm.

The roughness in a photoanode material may promote the scattering of incident photons during its interaction with the irradiation, thus affecting the photoactivity [33]. It can also be seen that most of the spheres were interfacial connected to each other, suggesting the coalescence mechanism [37] has been the driving force for the formation of a nanostructured C@TiO_2_ material [21].

Figure 3b shows the elemental analysis of the C@TiO_2_ (1:1) composite obtained from energy-dispersive X-ray spectroscopy (EDS), where Ti, O, C, N, and P atoms were detected. After calcination, the remaining carbon content in the C@TiO_2_ (1:1) sample is ca. 5.7 wt.% suggesting that increasing the agave:Ti volume ratio up to 8:1, an increase in carbon content should be expected, promoting the decrease in S_BET_ and total pore volume as a consequence of the porous framework blocking. In addition, it should be noted that the weight content of oxygen (ca. 56.7 wt.%) is 2.6 times higher than that observed for the titanium (ca. 21.4 wt.%). These values are clearly different compared to a TiO_2_ stoichiometry where the weight content of oxygen and titanium in this unit cell is ca. 45 wt.% and ca. 55 wt.%, respectively. This result suggests that oxygenated functional groups, mainly those associated with C atoms such as carbonyl and carboxyl groups from the lignin, cellulose, and sucrose structure contained in agave bagasse [25], would remain in the structure of the composite.

The images of Figure 3d–h obtained by SEM-EDS mapping showed a homogeneous distribution of the elements on the composite structure. This homogeneity agrees with the high reproducibility of E_bg_ results obtained from DR/UV-VIS analysis and also with the fact that some elements such as C, N and P, could be incorporated within the crystalline lattice of TiO_2_, playing the role of “p” or “n” type dopants [19]. In addition, the weight proportion of O to P was ca. 4.2, suggesting the formation of phosphate-like (PO_4^3−^_) or pyrophosphate-like (P_2_O_7^3−^_) groups that could be linked to 2 Ti^+4^ atoms forming Ti_2_P_2_O_7_ in line with the remarkable reduction in the E_bg_ of ca. 2.82 eV for C@TiO_2_ (1:1) composite compared to the commercial TiO_2_ of ca. 3.16 eV (Table 1).

**Figure 4:**
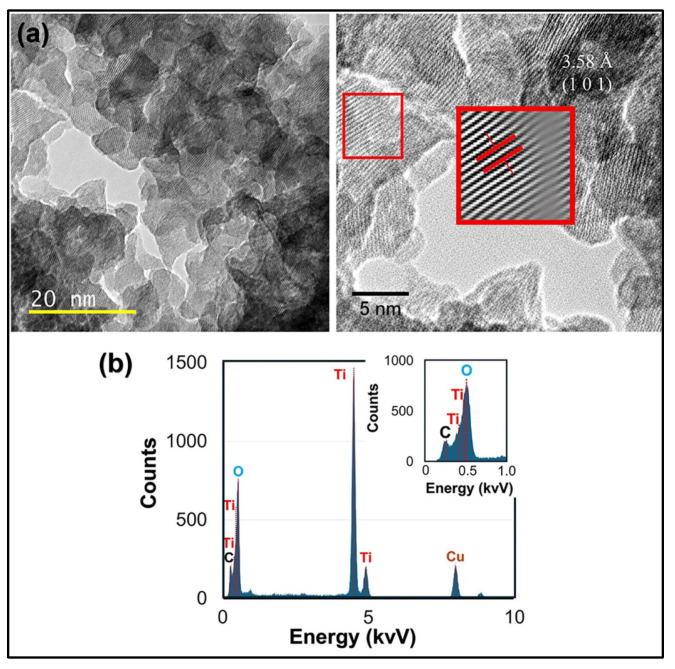
(**a**) HR-TEM image, (**b**) EDS spectrum of C@TiO_2_ (1:1) composite.

**Figure 5:**
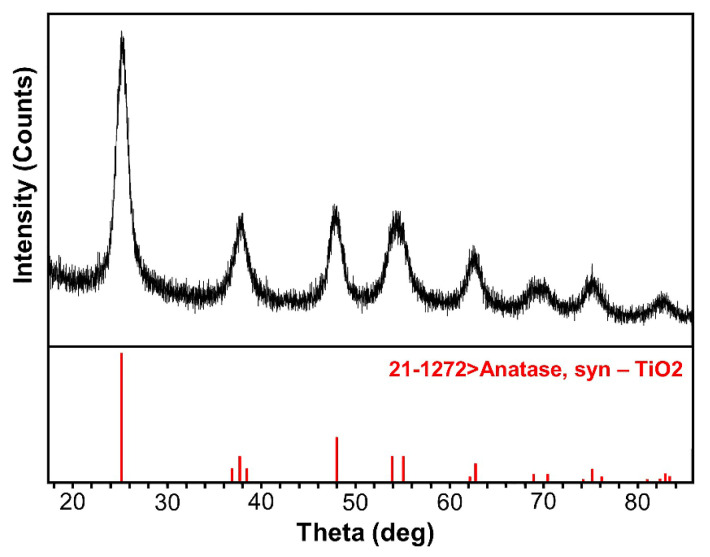
XRD patterns of calcined C@TiO_2_ composite (1:1).

Figure 4a shows the high-resolution transmission electron microscopy (HR-TEM) of the C@TiO_2_ (1:1) composite. It can be observed that a random agglomeration of nanoparticles, which is consistent with semiconductor nanomaterials prepared by a solvothermal process and calcined at a slightly lower temperature [38]. The average particle size of C@TiO_2_ (1:1) composite was ca. 7.1 nm ± 0.1. A magnification of the HR-TEM image confirms the formation of the anatase phase (101), where the spacing of the lattice fringes was around 0.358 nm, which corresponds to the spacing of the (101) facets of anatase [33]. Rutile phases were not detected in this analysis, indicating that the C@TiO_2_ (1:1) composite is mainly constituted by the anatase phase, as confirmed by the XRD patterns (Figure 5) discussed below.

Figure 4b shows the EDS spectrum of the C@TiO_2_ (1:1) composite. It can be observed that this sample is mainly composed of C, Ti, and O, which indicates that the carbonaceous material derived from the AHAB acts as an efficient templating agent for the synthesis of C@TiO_2_. However, despite the EDS analysis in Figure 4b showing carbon traces, it can be suggested that C atoms may influence the surface of the crystalline faces of TiO_2_ nanoparticles. This is inferred because the exposed surface of TiO_2_ showed a rougher surface [33], which agrees with the above discussion from SEM analysis. The surface roughness suggests that the C@TiO_2_ composite can present a lower scattering of incident photons, enhancing the light harvesting efficiency factor [19]. N and P were not detected on the surface of the composite, which suggested that these heteroatoms could be embedded within the spherical crystalline framework of TiO_2_. According to the SEM-EDS and HR-TEM analysis can be concluded that the morphological, structural, roughness, and compositional characteristics observed in C@TiO_2_ (1:1) could satisfactorily influence the photoelectric performance of this composite for its possible application in photovoltaic systems.

#### 2.1.5. XRD Analysis of C@TiO_2_ Composites

Figure 5 shows the X-ray diffraction (XRD) pattern of the C@TiO_2_ (1:1) composite. The JADE6 program permitted the identification of the main diffraction peaks at diffraction angles (2θ) equal to 25.4°, 38.1°, 47.7°, 54.4°, and 62.7°, corresponding to the (101), (004), (200), (211), and (204) crystallographic planes of the anatase phase of TiO_2_. The obtained pattern agrees with the standard PDF#21-1272 card for the anatase phase, in agreement with previous reports from Hua et al. [16] and Shen et al. [39].

A major proportion in the anatase phase for TiO_2_ in the C@TiO_2_ (1:1) composite will favor a greater absorption of both UV light and visible light. As reported by Yu et al. [40], this phenomenon can contribute to an effective capture and transport of induced photoelectrons on C-containing semiconductors for its possible application in the manufacture of carbon-derived photoelectrodes.

### 2.2. Photoelectrochemical Characterization of FTO/C@TiO_2_ Photoelectrodes in Dark and Under Light Irradiation

**Figure 6:**
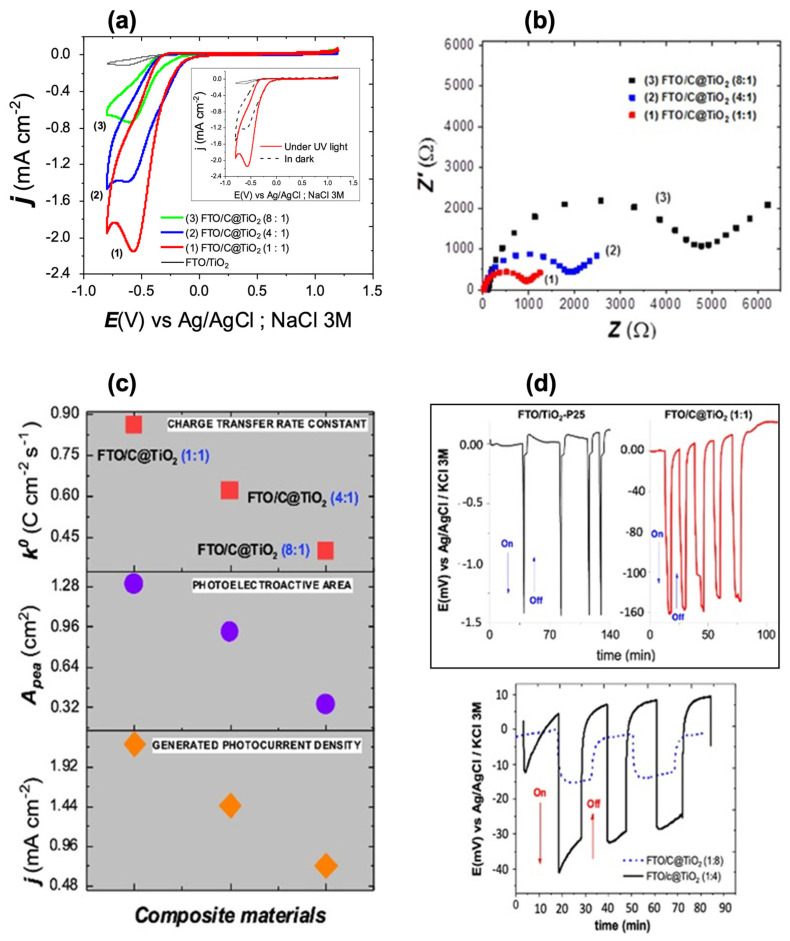
(**a**) CV responses for each photoelectrode. Inset shows the CV responses for the FTO/C@TiO_2_ photoelectrode with volume ratio (1:1) under UV irradiation (solid line) and dark conditions (dotted line); (**b**) Nyquist plots derived from EIS. EIS was carried out to 0.140 V (vs. RE), frequency range: 20 kHz to 1 mHz, width ca: 10 mV; (**c**) Graphics of photoelectrochemical parameters (J, A_pea_, and k°pct) for the photoelectrodes in presence of UV light; (**d**) Open-circuit potential variation curves, E(V) vs. time for FTO/TiO_2_-P25 and FTO/C@TiO_2_ (1:1 volume ratio) photoelectrodes.

The photoelectrochemical performance of FTO/C@TiO_2_ thin film photoelectrodes was studied by cyclic voltammetry (CV), electrochemical impedance spectroscopy (EIS), and variations of the open-circuit potential (OCP) employing a conventional three-electrode electrochemical cell in 0.1 M KCl as supporting electrolyte. In this case, FTO/C@TiO_2_ thin films acted as working photoelectrodes, Pt mesh as counter electrode, and the Ag/AgCl/NaCl 3 M system as reference electrode. The cyclic voltammetry study was performed with the following experimental conditions: a window potential from −0.8 to 1.2 V vs. Ag/AgCl/3 M NaCl, at a potential scan rate of 25 mV·s^−1^ in the cathodic direction starting at the open-circuit potential value (EOCP) under UV light irradiation and in the absence of light (in the dark). Figure 6a shows the CV response for the FTO/C@TiO_2_ photoelectrodes as a function of the volume ratio 1:1, 4:1, and 8:1 used for the synthesis of the C@TiO_2_ composites.

All CV responses showed the same cathodic potential peak nearby at E_pc_ = −0.60 V, attributed to the reduction of TiO_2_, indicating in turn the filling of surface states with electrons within the energy band gap of TiO_2_ [21]. The expected electrocatalytic phenomenon based on the cathodic overpotential (η_pc_/V) associated with the presence of C, N, and P together with TiO_2_ is also observed, suggesting the present photoelectrodes can be efficiently used as photoelectrocatalysts in redox-mediated processes.

It can also be seen from the CV curves from Figure 6a that the FTO/C@TiO_2_ (1:1) photoelectrode (curve 1) showed a higher photoelectroactivity performance, whose overpotential value (η_pc_ = −0.52 V) was up to 700 mV lower than the other two electrodes FTO/C@TiO_2_ (4:1) and FTO/C@TiO_2_ (8:1) with overpotential values of η_pc_ = −0.56 V and η_pc_ = −0.59 V, respectively. This fact can be attributed to a high homogeneity between TiO_2_ and the carbon atoms within the composite, which facilitates the application of a lower overpotential. It can also be suggested that this lower overpotential would promote the alignment of the Fermi levels of FTO/C@TiO_2_ (1:1) photoelectrodes, favoring the kinetics performance of interfacial charge transfer. The photoelectrochemical activation observed occurs because the C@TiO_2_ material is capable of absorbing and taking advantage of the UV irradiation in a better way than in the absence of carbon atoms. At the same time, the carbonaceous material acts as a harvester of photons and an electron reservoir in the composite, promoting a higher density of photocurrent, which leads to an additional contribution of pseudocapacitance and photoelectroactivity performance.

It should be mentioned that the photoelectroactivity performance is also favored by the presence of the other two heteroatoms, N and P. It is expected that the presence of P^+5^ and N^+3^ functional groups within the crystalline framework of the C@TiO_2_ composite played the role of electron-deficient and electron-rich doping agents [30], respectively, resulting in a higher concentration of photogenerated electrons in the electrochemical interface [41]. According to these results, it can be observed in Figure 6a that the photocurrent flux density (J) was three times higher in the FTO/C@TiO_2_ (1:1) photoelectrode than in the FTO/TiO_2_ photoelectrode composed of neat TiO_2_.

This behavior can be associated with the photoelectrochemical (PEC) efficiency of the electrode, which developed a photocurrent flux density of J = 2.2 mA·cm^−2^. The inset in Figure 6a shows the CV responses for FTO/C@TiO_2_ (1:1) under UV irradiation (solid line) and dark (dotted line) conditions. It can be seen that UV-driven radiation is responsible for generating a greater number of photoelectrons that are absorbed and accumulated at the electrochemical interface of the FTO/C@TiO_2_ (1:1) photoelectrode. Thus, the photoelectrode played the role of an electron donor, promoting a greater current density flow by applying a lower overpotential to the system.

On the contrary, the absence of light causes a low photoelectrochemical activity of the FTO/C@TiO_2_ (1:1) photoelectrode, which is reflected in the decrease of the current density flux. Also, the curves 2 and 3 in the CV analysis (Figure 6a) associated with FTO/C@TiO_2_ (4:1) and (8:1) photoelectrodes, respectively, presented a decrease in the photocurrent flux density as the amount of carbonaceous material in the composite increased. In other words, when carbon content on the photoelectrodes is increased, the photoactivity is passivated because the available photoelectroactive area and the charge transfer rate also decreased. To confirm this, the current density flux (J), the photoelectroactive area (A_pea_), the charge transfer rate constant (k°_pct_), and the charge transfer were estimated from the CV data and the resistance (R_ct_) from the Nyquist diagram. Table 2 lists the values of the photoelectrochemical parameters for the present FTO/C@TiO_2_ photoelectrodes.

On the other hand, Figure 6b shows the Nyquist diagrams from EIS measurements of the FTO/C@TiO_2_ photoelectrodes. The photoelectrode FTO/C@TiO_2_ (1:1) showed the lowest value of charge transfer resistance (R_ct_) when applying UV light (Table 2). This is attributed to a higher charge transfer rate at the electrochemical interface, which is favored by the adequate photoelectroactive area (A_pea_) and current density flux (J). The FTO/C@TiO_2_ (4:1) and (8:1) photoelectrodes showed 2 and 5 times higher values, respectively, in the charge transfer resistance (R_ct_) than the FTO/C@TiO_2_ (1:1) photoelectrode. This behavior was expected due to the photoelectrochemical passivation suffered by these photoelectrodes due to a greater amount of carbon in their structure.

Figure 6c shows a multigraph in terms of J, A_pea_, and k°_pct_ that allows for interpreting the influence of carbon content on the photoelectrochemical performance of TiO_2_ particles. In this case, the FTO/C@TiO_2_ (1:1) photoelectrode has the best photoelectrochemical behavior. This can be associated with a larger photoelectroactive area and superior interfacial heterogeneous kinetics, which promote an adequate flow of photoelectrons for their conversion to photocurrent. However, it is clear that this phenomenon was passivated in the other two FTO/C@TiO_2_ (4:1) and (8:1) photoelectrodes, which showed a significant decrease in the values of the photoelectrochemical parameters.

Figure 6d shows the variation of the open-circuit potential (OCP) of the three FTO/C@TiO_2_ photoelectrodes under UV light (365 nm) and dark conditions. The OCP measurements in Figure 6d allow the evaluation of both the difference between the apparent Fermi level of the working photoelectrode and the electron accumulation at the photoelectroactive surface [42]. As reported by Yu and Wang [43], the OCP value of TiO_2_ photoelectrodes depends on: (i) the transfer rate of the charge carriers from the surface to the internal structure of TiO_2_, (ii) the recombination rate of the photoinduced electron–hole pairs, and (iii) the reactions of the surface electrons with the photooxidized species in the electrolyte. It can be seen that when the photoelectrodes were irradiated with UV light, labeled as the ON mode, it is evident that the OCP of the photoelectrodes became more negative, especially the FTO/C@TiO_2_ (1:1) photoelectrode, which presented the most negative value of the three films. Thus, at steady-state conditions of interfacial charges, a greater capture and accumulation of photoelectrons was achieved at the electrochemical interface of the photoelectrode FTO/C@TiO_2_ (1:1). Subsequently, upon interrupting irradiation, labeled as the OFF mode, the OCP became positive between each electrode charge-discharge until reaching the equilibrium of the OCP value in the dark [21,44]. The rate of change of the OCP value of the FTO/C@TiO_2_ (1:1) photoelectrode was the fastest and more negative, suggesting that the rate of accumulation and load transfer was more efficient, requiring less time to raise the photon-driven potential to a specific value compared to TiO_2_-P25. The present results agree with sensitized carbon quantum dot TiO_2_-based photoelectrodes using orange peels as a carbon source [22]. Also, an enhancement in the steady-state condition of the photon-driven potential was observed, indicating that the C@TiO_2_ (1:1) film presented a lower rate of recombination between the photoinduced electron–hole pair.

The results demonstrate that the material prepared with an agave:Ti volume ratio of 1:1 yields the best optical and photoelectrochemical characteristics. In contrast, increasing the volume of AHAB in the synthesis reactor did not produce the same photoelectrochemical performance in the resulting composite because an excess of carbon promotes the formation of a large amount of amorphous carbon unable to be integrated adequately with the P and N heteroatoms into the semiconductor to substantially improve its optical and electrochemical properties. Therefore, this work suggests that increasing the AHAB ratio is neither necessary nor beneficial because irregular carbon deposits are formed on the TiO_2_ with a detrimental effect on its photoelectrochemical properties.

### 2.3. Performance of FTO/C@TiO_2_ Photoelectrodes in Solar Cells

**Figure 7:**
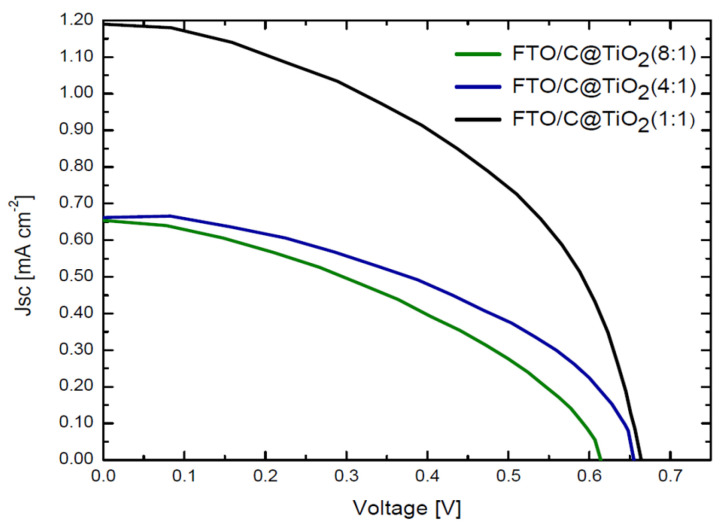
I–V curves of FTO/C@TiO_2_ photoanodes in solar cells.

In accordance with the excellent optical and photoelectrochemical properties shown by the photoelectrodes FTO/C@TiO_2_, the C@TiO_2_ composites were used as photoanodes in the fabrication of organic waste-derived solar cells (OWSC), here labeled as OWSC 1, OWSC 2 and OWSC 3, for the composites with volume ratios (1:1), (4:1) and (8:1), respectively. Once the corresponding photovoltaic cells were assembled, they were evaluated within a SciSun-300 solar simulator, and the current and voltage data from the performance of the FTO/C@TiO_2_ photoanodes were obtained in triplicate. These results are shown in Figure 7.

All photovoltaic solar cells presented the characteristic I-V curve shape of a photovoltaic device. The I–V curves permit estimation of the main parameters of a photovoltaic cell, such as open-circuit voltage (VOC), maximum power (W_max_), the photovoltaic energy conversion (PEC) factor, also called conversion efficiency (η), and the short-circuit current multiplied by the cell area to be presented as short-circuit current density (J_sc_). A summary of these results is listed in Table 3.

The comparison between the standard deviation and the average values yields the following percentual coefficients of variation (CV’s): 0.2–3.0%, 4.6–13%, 4.3–6.7%, and 6.3–10.5% for V_oc_, J_sc_, the fill factor, and η, respectively. The CV’s indicate that for most of the variables, the experimental parameters obtained for the assembled solar cells are statistically acceptable because they showed ≤10% variation around the average value.

The solar cells constructed with the FTO/C@TiO_2_ (1:1) composite presented the best performance with a conversion efficiency of 0.375% compared to the other FTO/C@TiO_2_ (4:1 and 8:1) photoelectrodes with values of 0.160% and 0.194%, respectively. This is attributed to the activation of the solar device, which, after the absorption of photons, is able to promote the excitation of photo-generated electrons at the interface of the C@TiO_2_ (1:1) thin film [45]. As suggested above, this boosted optoelectronic performance can be attributed to the presence of heteroatoms such as N and P, besides C, within the crystalline framework of TiO_2_ [22]. In agreement with a higher energy band gap (Table 1) and poor electrochemical behavior (Table 2) discussed above, the increase in the amount of AHAB for the preparation of the C@TiO_2_ (with a volume ratio of 4:1 and 8:1) composites caused a passivation of the photoelectrochemical properties in the electrodes with a detrimental effect on the photoefficiency of solar-to-electric energy (Table 3).

Table 4 shows a general comparison of the results obtained in terms of the synthesis parameters and photoefficiency for the present OWSC 1 solar cell against other organic solar cells reported [15,46,47,48,49,50,51].

Table 4 shows that the synthesis process of the C@TiO_2_ composites reported in the present work seems to be more efficient than other works relative to time (t_syn_ = 5 h) and temperature (T_syn_ = 350 °C). The photoefficiency conversion factor (PEC) reported for the present work is ca. 0.38% for the solar cell OWSC 1. This value is 12.7 times higher than that of the solar cell composed of FTO/TiO_2_ [46]. Other studies included in Table 4 are also characterized for the use of biomass waste to generate electrodes coupled to photovoltaic cells. For instance, Chen et al. [15] reported a photoefficiency factor of 1.44% for the solar cell prepared by combining CQDs obtained from sugarcane bagasse to sensitize and TiO_2_ nanotubes (CQDs/TNTAs) as photoelectrodes for solar cells, but the time and temperature conditions used are clearly higher than those reported in the present work.

Maiaugree et al. [47] published the use of mangosteen peel waste to manufacture carbon (MPC) counter electrodes, employing the carbonized shell product and the biomass dye as a photosensitizer for TiO_2_. The best photovoltaic cell configuration achieved an energy conversion efficiency of up to 2.63%, attributed in part to the photosensitizer, but also to the synergy between the carbon counter electrode and the electroconductive polymers. However, the synthesis of TiO_2_ involved the thermal decomposition of TiCl_4_ at 550 °C and the pyrolysis of mangosteen peels at 850 °C for 2 h.

Meneghetti et al. [48] reported the performance of a DSSC-type photovoltaic cell using biomass waste byproducts from the winemaking process, utilizing the extracted dye rich in phenols, tannins, and phthalocyanines, playing the role of photosensitizers for TiO_2_. The best-performing DSSC shows an energy conversion efficiency of 0.45%, attributed to the extracted organic dye. Ashok et al. [49] extracted natural photoactive dye from Yaca organic waste to be used as a photosensitizer for nanostructured TiO_2_. These DSSCs exhibited a photoconversion efficiency of ca. 1.1%. In the same trend, Hosseinnezhad et al. [50] extracted a separate photosensitizing dye from a mixture of agri-food waste composed of eggplant, cherries, and red grapes. The best-performing DSSC showed an energy conversion efficiency of 1.49% using a TiO_2_ photoanode photosensitized with the extracted dye and a platinum counter electrode. Finally, Xu et al. [51] investigated the configuration and photovoltaic performance of a dual system based on bio-waste. The DSSCs showed an approximate energy conversion efficiency of 1.50%, showing the valorization of waste in the field of energy conversion, but the material used in this work was prepared at high temperature (800 °C). In summary, it is clear that some of these photoefficiencies are higher than the value reported in the present work, but it is also clear that the present methodology uses more eco-friendly experimental conditions.

### 2.4. Final Discussion

It is worth noting that although we do not present XPS results in this work, it should be emphasized that energy-dispersive X-ray spectroscopy (EDS) is considered a surface analysis technique totally valid to demonstrate the presence of atoms on surfaces. Therefore, the EDS analysis and SEM-EDS mapping of Figure 3 clearly show that the C, N, and P heteroatoms are homogeneously distributed in the C@TiO_2_ (1:1) composite, suggesting that C, P, and even N at a much lower atomic concentration, are incorporated not only as surface-decorating atoms but also possibly intercalated within the TiO_2_ crystal structure reported in previous studies of C-doped TiO_2_ [33].

**Figure 8:**
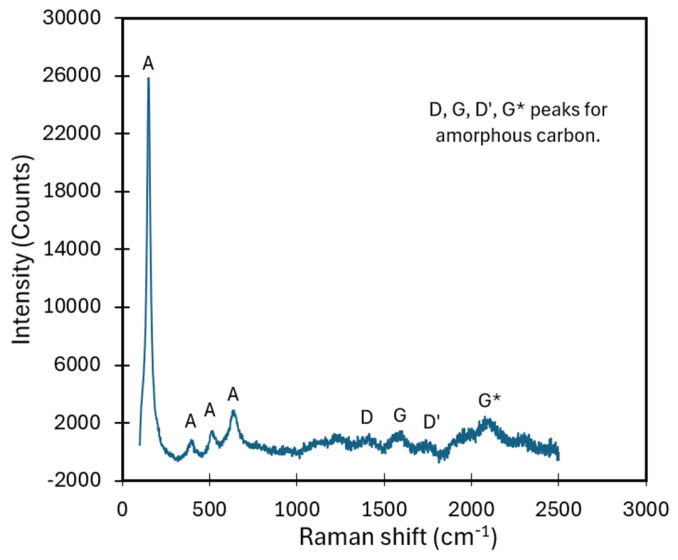
Raman spectra of C@TiO_2_ (1:1) composite.

However, for a better description of the carbon phase in the present study, Figure 8 shows the baseline-fitted Raman spectrum of the C@TiO_2_ (1:1) composite. Firstly, this figure shows the typical fingerprint of the TiO_2_ anatase phase [52] with the bands at 151 (E_g_ mode), 393 (B_1g_ mode), 513 (A_1g_ mode), and 652 cm^−1^ (E_g_ mode). These modes are in good agreement with the almost pure anatase phase observed in the XRD pattern discussed above (Figure 5).

The characteristic defects (D) and graphitic (G) bands of carbon-based materials [53] cannot be clearly appreciated in Figure 8, probably due to the low carbon content (ca. 5.7 wt.%) as discussed above from the EDS analysis in Figure 3b. However, the features observed at 1409 (D band), 1590 (G band), and 1651 cm ^−1^ (D′ band) can be attributed to amorphous carbon [53] or to the presence of carbon quantum dots in the C@TiO_2_ (1:1) composite. Another amorphous peak at 2073 cm^−1^, indicated as G*, can be attributed to an overtone of the D-peak [53]. On the other hand, the differences found between the experimental atomic weight proportions for Ti and O atoms observed by EDS compared to the theoretically expected values discussed above suggest that certain elements may be incorporated into the semiconductor structure as dopants. Multiple reports of this have been published in the literature. For instance, previous experimental results and theoretical calculations from our group [33] have shown that the incorporation of ca. 1 wt.% C into the TiO_2_ lattice is responsible for an increase in the density of states of the semiconductor due to the contribution of the C 2p orbitals. The formation of this heterojunction would not only promote a greater charge separation but also a decrease in the energy band gap, as observed in the present composites. Our group demonstrated [33] that 350 °C is a suitable calcination temperature for the intercalation of low C content within the crystal structure of TiO_2_ in the C@TiO_2_ composite, which can be extrapolated to other heteroatoms. Thus, it can be suggested that the intercalation of C, N, and P heteroatoms is responsible for the significant decrease in the energy band gap observed in the present samples. Consequently, the intercalated C, N, and P atoms solved, in a certain manner, the limitations of TiO_2_, mainly those related to charge transfer kinetics, improving the quantum efficiency of the semiconductor.

However, at the same time, it should be noted that the remaining oxygen atoms linked to carbon atoms may also play the role of charge traps. For instance, oxygenated groups linked to carbon atoms can form surface complexes with TiO_2_ [54] or Ti-containing biogenic silica [55], being responsible for the formation of a non-stoichiometric titanium oxide (Ti_3_O_5_) with a partial reduction of the oxidation state of Ti from Ti^+4^ to Ti^+3<δ<+4^ [54]. Thus, an excess of oxygen-containing groups in the organic solar cell could induce a detrimental effect on the overall conversion efficiency of the solar cell. Thus, this important effect will be incorporated in future work. Finally, it should be noted that carbon phase in the present organic-based solar cell system is not an inert coating layer but an active component participating in charge transfer and chemical reactivity [56,57].

## 3. Materials and Methods

### 3.1. Materials

All the following reagents were analytical grade and used without further purification. Titanium (IV) isopropoxide (>97%, high-purity), terpineol (≥95%), ethyl cellulose (48%), potassium ferricyanide (≥99%), were purchased from Sigma-Aldrich (Burlington, MA, USA), and absolute ethanol (99.99%, ultra-high purity), sodium chloride (100%), potassium chloride (99.3%), nitric acid (65.9%), acetone (99.97%, ultra-high purity) from Fermont (Monterrey, Nuevo Leon, Mexico). Commercial TiO_2_-P25 was purchased from Evonik (Essen, Germany, ex-Degussa). Hydrochloric acid (0.1 M, standard, from J. T. Baker, Radnor, PA, USA). The bagasse from agave Tequilana Weber Var was collected from the waste generated at the Casa Herradura tequila company located in Amatitán (Jalisco, Mexico). All synthesis and characterization used deionized water (18 MΩ·cm), when needed.

### 3.2. Synthesis of C@TiO_2_ Composites

The software Design-Expert 7.0 (Minneapolis, MN, USA) was used to plan the microwave-assisted solvothermal synthesis of the C@TiO_2_ composites considering a previous work [21]. Acid hydrolysate of agave bagasse (AHAB) was used a carbon and heteroatoms source. The suspensions were prepared with the following volume ratios: 1:1, 4:1, and 8:1 (*v*/*v*) of carbon precursor (agave) and titanium alkoxide (Ti(isop)_4_), respectively. This mixture, denoted as agave:Ti, was placed into a microwave vessel with 10 mL of ethanol. The vessel was tightly sealed, placed in the microwave oven, and the temperature increased from ambient to 130 °C in 5 min with a power of 400 W. This temperature was maintained for 30 min. The resulting suspension was cooled down and decanted. The obtained solid was centrifuged and washed several times with absolute ethanol and then dried at 80 °C for 4 h in an oven. Finally, the samples were calcined at 350 °C for 5 h. The so-prepared compounds have been labeled as C@TiO_2_ (1:1), C@TiO_2_ (4:1), and C@TiO_2_ (8:1), highlighting that the numbers between parentheses indicate the volume ratios of agave:Ti used for the synthesis.

### 3.3. Characterization of C@TiO_2_ Composites

The analysis of the morphology, structure, and surface composition of the samples was carried out using a dual-beam scanning electron microscopy (FIB/SEM, Thermo-Fischer Scientific, Waltham, MA, USA), a high-resolution transmission electron microscope (HRTEM, JEOL 200 CX, Peabody, MA, USA), and energy-dispersive X-ray spectroscopy (EDS, FEI-Helios Nanolab 600, Hillsboro, OR, USA), respectively.

The crystalline phases of TiO_2_ were determined from the X-ray diffraction (XRD) patterns obtained by a SmartLab diffractometer from RIGAKU (Akishima-Shi, Japan) with Cu Kα radiation (λ = 1.5418 Å). The scanning range was from 20°–80° for C@TiO_2_ composites. The scanning speed was 5°·min^−1^ with a scanning step of 0.01°.

The diffuse reflectance UV-vis spectra (DR/UV-VIS) of TiO_2_ and C@TiO_2_ samples were measured in a Cary 6000i UV–Vis-NIR spectrophotometer from Agilent (Santa Clara, CA, USA) equipped with a diffuse reflectance accessory. The incident beam was collimated, and reflected light was captured by an integrating sphere.

The BET surface area, pore volume and pore size distribution were determined from the data of the adsorption-desorption N_2_ isotherms at −196 °C using an ASAP 2020 equipment from Micromeritics (Norcross, GA, USA).

Elemental analysis (C, N, H) was carried out using a COSTECH 4010 (Raleigh, NC, USA) elemental analyzer. The equipment was calibrated with 5 points for the three elements, using acetanilide as a standard containing 71.09% C, 6.71% N and 10.36% H. For this, 5 to 20 mg of sample were weighed into a tin capsule and placed in the autosampler for analysis.

The elemental composition of the acid-hydrolysate agave bagasse (AHAB) sample was determined after processing the AHAB with nitric and sulfuric acid, and subsequently the samples were analyzed in an inductively coupled plasma emission spectrophotometer (ICP-OES), Varian model 730 ES (Palo Alto, CA, USA).

The Raman spectrum of the calcined C@TiO_2_ composite (1:1) powder was recorded in ambient conditions using an InVia Microraman Renishaw spectrometer (Wotton-under-Edge, Gloucestershire, UK) with a wavelength laser of 532 nm. The spectra were collected under an optical microscope with a ×50 long working distance objective, and each spectrum was recorded with an integration time of 10 s.

### 3.4. Fabrication and Study of FTO/C@TiO_2_ Photoelectrodes

The FTO/C@TiO_2_ working photoelectrodes were fabricated through the screen-printing method. For this, 2.0 × 2.0 cm glasses of fluorine-doped tin oxide (FTO) were used as photocurrent collector substrates, which were exhaustively washed with isopropanol and sonicated for 30 min before the deposition of the C@TiO_2_ materials [58]. A paste was obtained by constant stirring of a mixture of 50 mg C@TiO_2_ powder dispersed in a terpineol and ethyl cellulose solution in acetone with a weight ratio of 1:2:7, respectively. Consecutively, the FTO electrode was coated with a thin film of the resulting paste. The film on the glass was sintered at 450 °C for 30 min under an air atmosphere. The coated area of the photoelectrode was 2.25 cm^2^. A conventional three-electrode cell was implemented using FTO/C@TiO_2_ as working photoelectrodes using the hybrid C@TiO_2_ samples prepared from 1:1, 4:1, and 8:1 volume ratios of agave:Ti. Pt mesh was used as the counter electrode, and the Ag/AgCl/NaCl 3 M system as the reference electrode. The sensitized FTO/C@TiO_2_ photoanodes were positioned towards the side of the cell on which the light irradiation is incident. The irradiation was done at 365 nm with an Hg-based UV Pen Ray lamp (12.2 W·m^−2^) from Analytik Jena (Tewksbury, MA, USA), in a quartz tube. The electrochemical and photoelectrochemical measurements were performed with a potentiostat/galvanostat model VSP SAS Bio-Logic (Seyssinet-Pariset, Auvergne-Rhone-Alpes, France) controlled by EC-Lab software V 10.23 [22].

The cyclic voltammetry measurements were performed in a 0.1 M KCl solution at room temperature. The scan started at the open-circuit potential in a cathodic direction until a reversal potential of E_λ_ = −0.8 V, then in an anodic direction until a reversal potential of E_λ_= 1.2 V vs. Ag/AgCl/NaCl 3 M, at a potential scan rate of 25 mV·s^−1^. The electrochemical impedance spectroscopy measurements were carried out at 0.140 V against Ag/AgCl/NaCl 3 M, within a frequency range from 20 kHz–1 mHz using ca. 10 mV.

### 3.5. Behavior of FTO/C@TiO_2_ Photoanodes in Solar Cells

The FTO/C@TiO_2_ photoanodes were preliminary evaluated in the assembly organic waste-derived solar cells (OWSCs). These photoanodes were labeled as FTO/C@TiO_2_ (1:1), (4:1), (8:1). The design and configuration of the photovoltaic cells is the following. FTO/C@TiO_2_ as working electrode and a platinum film was selected as the counter electrode. SurlynTM 1720 polymer frame was used as an optically transparent separator between the working and the counter electrodes. The supporting electrolyte containing the I¯/I_3_ pair from Iodolyte HI-30 solution (Solaronix, Aubonne, Switzerland), was injected, and finally a silver contact was added. The respective current and voltage (I-V) measurements were performed using a SciSun-300 solar simulator from SCIENCETECH (London, ON, Canada) with a Xenon short-arc lamp (UXL-150S0) in conditions of air mass AM 1.5 (1000 W·m^−2^) with a voltage-current meter from Keithley (Solon, OH, USA) model 2400.

## 4. Conclusions

The synthesis of hybrid organic–inorganic materials C@TiO_2_ using acid hydrolysate from agave bagasse by a microwave-assisted solvothermal process is reported. A carefully characterization of materials, including textural, structural, compositional, and optical properties suggests that the C@TiO_2_ composite with agave:Ti volume ratio equal to (1:1) exhibited a major optical behavior and to be used as photoactive devices. TiO_2_-based nanoparticles presented carbon, nitrogen, and phosphorus doping, which were beneficial to improve the excitation of photoelectrons with a decrease in the energy band gap from 3.2 eV for TiO_2_-P25 to 2.8 eV for the C@TiO_2_ (1:1) composite.

The synergistic effect promoted by the presence of C, P, and N also reflects a better charge transfer kinetic capacity value of 0.86 C·cm^−2^·s^−1^ resulting in a higher current density flow of 2.2 mA·cm^−2^ in the photoelectrochemical studies of this composite. The photoanode composed by FTO/C@TiO_2_ prepared from the volume ratio 1:1 was evaluated in a photovoltaic solar cell showing representative a solar-to-electric conversion with a maximum of 0.38% efficiency which is up to 12.7 times higher than the photoanode composed by neat TiO_2_.

The present results contribute to the state-of-the-art of organic–inorganic thin film photoelectrodes for the sustainable synthesis of 3rd generation solar cells using bagasse-derived wastes as an efficient carbon source for the enhancement of the optical properties of photoactive semiconductors. Compared with other similar solar cells devices, it can be concluded that the use of acid hydrolysate agave bagasse (AHAB) as a template permits to prepare in a single, low-cost, and ecofriendly manner N- and P-containing C@TiO_2_ composites with a high potential to be used for the fabrication of organic waste-derived solar cells. However, it is also important to highlight that bio sourced materials like bagasse can have different batch to batch compositions. The performance of solar cells can be remarkable affected as the result of the composition variability in different batches of agave bagasse. Thus, it is recommended to perform a careful characterization of the biomass source to stablish the correct ranges of works for organic solar cells as a function of the chemical components of biomass waste.

## Figures and Tables

**Table 1:** Optical and textural properties of C@TiO_2_ and TiO_2_-P25.

Sample	E_bg_ ^a^ (eV)	Red-Shift λ (nm)	BET ^c^ (m^2^·g^−1^)	Total Pore Volume ^d^ (cm^3^·g^−1^)	Pore Diameter ^e^ (nm)
TiO_2_-P25	3.22 ^b^	385	50 ^f^	-	-
C@TiO_2_ (1:1)	2.92 ± 0.03	424	31	0.029	1.8
C@TiO_2_ (4:1)	3.01 ± 0.01	411	56	0.046	1.6
C@TiO_2_ (8:1)	2.85 ± 0.05	435	48	0.040	1.7

^a^ Energy band gap values of C@TiO_2_ composites estimated from the linear regressions from Figure 1b–d. ^b^ Optical data from TiO_2_-P25 taken from reference [19]. ^c^ Surface area estimated from BET equation. ^d^ Total pore volume estimated at P/P_o_ = 0.97. ^e^ Average pore diameter estimated from BJH (Barrett–Joyner–Halenda) equation. ^f^ Surface area from TiO_2_-P25 taken from reference [19].

**Table 2:** Photoelectrochemical parameters for FTO/C@TiO_2_ photoelectrodes.

Photoelectrode	J (mA·cm^−2^)	A_pea_ ^a^ (cm^2^)	k°_pct_ ^b^ (C·cm^−2^·s^−1^)	R_ct_ (Ω)
FTO/C@TiO_2_ (1:1)	2.20 ± 0.08	1.30 ± 0.02	0.86 ± 0.01	433
FTO/C@TiO_2_ (4:1)	1.45 ± 0.01	0.92 ± 0.03	0.62 ± 0.06	872
FTO/C@TiO_2_ (8:1)	0.73 ± 0.02	0.35 ± 0.01	0.40 ± 0.01	2186

^a^ A_pea_ was calculated from Equation (S1) (SI) using the experimental values of i_p_, and then J (current density) was calculated from i_p_/A_pea_. ^b^ k°_pct_ was calculated from Equation (S2) (SI). In both estimations, a 5 mM K_4_[Fe(CN)_6_] + 0.1 M KCl solution at a scan rate from 20 to 160 mV·s^−1^ under UV irradiation was used.

**Table 3:** Experimental parameters obtained from the assembled solar cell.

Name	Configuration of Photoanodes	Volume Ratio Agave:Ti	V_oc_ [V]	J_sc_ [mA]	Fill-Factor (a.u)	η [%]
OWSC 1	FTO/C@TiO_2_	1:1	0.664 ± 0.004	1.19 ± 0.09	0.47± 0.02	0.38 ± 0.04
OWSC 2	FTO/C@TiO_2_	4:1	0.614 ± 0.001	0.65 ± 0.03	0.40 ± 0.02	0.16 ± 0.01
OWSC 3	FTO/C@TiO_2_	8:1	0.655 ± 0.020	0.66 ± 0.09	0.45 ± 0.03	0.19 ± 0.02

**Table 4:** Comparison of experimental parameters used for the fabrication of organic solar cells using carbon-derived photoelectrodes.

Solar Cell	Configuration of Photoelectrodes	t_syn_ (h)	T_syn_ (°C)	η [%]	Reference
OWSC 1	FTO/C@TiO_2_ (1:1)	5	350	0.38	This work
QDSSCs	FTO/CQDs/TNTAs	6_step1_ 8_step2_	100_step1_ 450_step2_	1.44	[15]
Solar cell	FTO/TiO_2_-bare	-	-	0.03	[46]
DSSC	FTO/PDOT-PSS/mangosteen peels	2	850_(Argon)_	2.63	[47]
	FTO/TiO_2_/organic dye-Wine	0.5	400_step1_ 600_step2_	0.45	[48]
	FTO/TiO_2_/organic dye-Yaca	-	-	1.07	[49]
	FTO/TiO_2_/organic dye-Natural	-	-	1.49	[50]
	FTO/TiO_2_/BND	1	800	1.50	[51]

## Data Availability

The original contributions presented in this study are included in the article/Supplementary Materials. Further inquiries can be directed to the corresponding authors.

## Supplementary Materials

The following supporting information can be downloaded at: https://www.mdpi.com/article/10.3390/molecules31132399/s1, Table S1. CHONS analysis of agave bagasse acid hydrolysate (AHAB). Table S2. Compositional analysis of agave bagasse acid hydrolysate (AHAB). Table S3. Elemental analysis of agave bagasse acid hydrolysate (AHAB) from inductively coupled plasma-optical emission spectroscopy (ICP-OES). Figure S1. Elemental composition of powders of Agave Bagasse (PAB). Equation (S1): $i_{p}=2.69x10^{5}n^{3/2}ApeaC_{0}D_{redox}^{1/2}\upsilon^{1/2}$. Definition of the electrochemical parameters in Equation (1). i_p_: the current intensity of peak, n: electrons transferred in the electrochemical interface, A_pea_: photoelectroactive area of working electrode, C_o_: molar concentration of redox probe, K_4_[Fe(CN)_6_], D_redox_: diffusion coefficient of redox probe, K_4_[Fe(CN)_6_], v: scan rate on electrochemical system. Equation (S2): $E_{p}=\left(\frac{2.303RT}{n*k_{pct}^{0}*F}\right)logv+C$. Definition of the electrochemical parameters in Equation (2): E_p_: value potential of the peak, R: universal constant of ideal gases, T: temperature, n: electrons transferred in the electrochemical interface, k°_pct_: electrochemical kinetic, F: Faraday constant, ν: scan rate on electrochemical system, C: molar concentration of redox probe, K_4_[Fe(CN)_6_].

## References

[1] King R.R., Bhusari D., Larrabee D., Liu X.Q., Rehder E., Edmondson K., Cotal H., Jones R.K., Ermer J.H., Fetzer C.M. (2012). Solar cell generations over 40% efficiency. Prog. Photovolt..

[2] Sharma K., Sharma V., Sharma S.S. (2018). Dye-Sensitized Solar Cells: Fundamentals and Current Status. Nanoscale Res. Lett..

[3] O’Regan B., Grätzel M. (1991). A low-cost, high-efficiency solar cell based on dye-sensitized colloidal TiO_2_ films. Nature.

[4] Wu J., Lan Z., Lin J., Huang M., Huang Y., Fan L., Luo G., Lin Y., Xiea Y., Weia Y. (2017). Counter electrodes in dye-sensitized solar cells. Chem. Soc. Rev..

[5] Wang Y.Y., Chen Y.X., Barakat T., Zeng Y.J., Liu J., Siffert S., Su B.L. (2022). Recent advances in non-metal doped titania for solar-driven photocatalytic/photoelectrochemical water-splitting. J. Energy Chem..

[6] Ohtani B. (2013). Titania hotocatalysis beyond ecombination: A critical review. Catalysts.

[7] Piatkowska A., Janus M., Szymanski K., Mozia S. (2021). C-,N- and S-doped TiO_2_ photocatalysts: A review. Catalysts.

[8] Wu C., Wang K., Batmunkh M., Bati A.S.R., Yang D., Jiang Y., Hou Y., Shapter J.G., Priya S. (2020). Multifunctional nanostructured materials for next generation photovoltaics. Nano Energy.

[9] Bautista-López J.A., Díaz-Ponce A., Rangel-Méndez J.R., Cházaro-Ruiz L.F., Mumanga T.J., Olmos-Moya P., Vences-Álvarez E., Pineda-Arellano C.A. (2023). Recent progress in organic waste recycling materials for solar cell applications. Environ. Sci. Pollut. Res..

[10] Ma P., Lu W., Yan X., Li W., Li L., Fang Y., Yin X., Liu Z., Lin Y. (2018). Heteroatom tri-doped porous carbon derived from waste biomass as Pt-free counter electrode in dyesensitized solar cells. RSC Adv..

[11] Urbina A. (2020). The balance between efficiency, stability and environmental impacts in perovskite solar cells: A review. J. Phys. Energy.

[12] Liu H., Xie Y., Wei P., Wang W., Chen H., Geng C., Qiang Y. (2020). Interface optimization of hole-conductor free perovskite solar cells using porous carbon materials derived from biomass soybean dregs as a cathode. J. Alloys Compd..

[13] Dasari K.K., Gumtapure V. (2019). Activated carbon-based dye-sensitized solar cell for development of highly sensitive temperature and current sensor. Mater. Res. Express.

[14] Wang C.L., Meng F.N., Wang T.H., Ma T.L., Qiu J.S. (2014). Monolithic coal-based carbon counter electrodes for highly efficient dye-sensitized solar cells. Carbon.

[15] Chen Q., Lin G., Meng L., Zhou L., Hu L., Nong J., Li Y., Wang J., Hu K., Yu Q. (2020). Enhanced photoelectric performance of TiO_2_ nanotubes sensitized with carbon dots derived from bagasse. Chem. Phys. Lett..

[16] Hua L., Yin Z., Cao S. (2020). Recent advances in synthesis and applications of Carbon-doped TiO_2_ nanomaterials. Catalysts.

[17] Saharudin K.A., Sreekantan S., Lai C.W. (2014). Fabrication and photocatalysis of nanotubular C-doped TiO_2_ arrays: Impact of annealing atmosphere on the degradation efficiency of methyl orange. Mater. Sci. Semicond. Process..

[18] Basavarajappa P.S., Patil S.B., Ganganagappa N., Reddy K.R., Raghu A.V., Reddy C.V. (2020). Recent progress in metal-doped TiO_2_, non-metal doped/codoped TiO_2_ and TiO_2_ nanostructured hybrids for enhanced photocatalysis. Int. J. Hydrogen Energy.

[19] Matos J., Atienzar P., Garcia H., Hernandez-Garrido J.C. (2013). Nanocrystalline carbon-TiO_2_ hybrid hollow spheres as possible electrodes for solar cells. Carbon.

[20] Xu Y.Y., Lu S.Q., Zheng Y.Z., Fang H.B., Tao X., Chen J.F. (2015). Visible-light driven C@TiO_2_ porous films: Enhanced photoelectrochemical and photoelectrocatalytic performance. Catal. Commun..

[21] Rangel-Mendez J.R., Matos J., Chazaro-Ruiz L.F., Gonzalez-Castillo A.C., Barrios-Yanez G. (2018). Microwave-assisted synthesis of C-doped TiO_2_ and ZnO hybrid nanostructured materials as quantum-dots sensitized solar cells. Appl. Surf. Sci..

[22] Olmos-Moya P.M., Velazquez-Martinez S., Pineda-Arellano C., Rangel-Mendez J.R., Chazaro-Ruiz L.F. (2022). High added value functionalized carbon quantum dots synthetized from orange peels by assisted microwave solvothermal method and their performance as photosensitizer of mesoporous TiO_2_ photoelectrodes. Carbon.

[23] Nair R., Rishyavandhan V., Kumar M.A.A., Dandeker N., Velvizhi G. (2026). Unravelling the potential of biochar derived activated carbon functionalized TiO_2_ for photocatalytic degradation of congo red via RSM optimization. Chem. Sel..

[24] Wang C., Yu R. (2022). Highly efficient visible light photocatalysis of tablet-like carbon-doped TiO_2_ photocatalysts via pyrolysis of cellulose/MIL-125(Ti) at low temperature. J. Solid State Chem..

[25] Velazquez-Jimenez L.H., Pavlick A., Rangel-Mendez J.R. (2013). Chemical characterization of raw and treated agave bagasse and its potential as adsorbent of metal cations from water. Ind. Crops Prod..

[26] Kudr J., Richtera L., Xhaxhiu K., Hynek D., Heger Z., Zitka O., Adam V. (2017). Carbon dots based FRET for the detection of DNA damage. Biosens. Bioelectron..

[27] Chen T.H., Tseng W.L. (2017). Self-assembly of monodisperse carbon dots into high-brightness nanoaggregates for cellular uptake imaging and Iron(III) sensing. Anal. Chem..

[28] Han L., Liu S.G., Dong J.X., Liang J.Y., Li L.J., Li N.B., Luo H.Q. (2017). Facile synthesis of multicolor photoluminescent polymer carbon dots with surface-state energy gap-controlled emission. J. Mater. Chem. C.

[29] Cheng J., Wang C.F., Zhang Y., Yang S.Y., Chen S. (2016). Zinc ion-doped carbon dots with strong yellow photoluminescence. RSC Adv..

[30] Hu C., Li M.Y., Qiu J.S., Sun Y.P. (2019). Design and fabrication of carbon dots for energy conversion and storage. Chem. Soc. Rev..

[31] Holá K., Sudolská M., Kalytchuk S., Nachtigallová D., Rogach A.L., Otyepka M., Zbořil R. (2017). Graphitic nitrogen triggers red fluorescence in carbon dots. ACS Nano.

[32] Peng C., Wang W., Zhang W., Liang Y., Zhuo L. (2017). Surface plasmon-driven photoelectrochemical water splitting of TiO_2_ nanowires decorated with Ag nanoparticles under visible light illumination. Appl. Surf. Sci..

[33] Matos J., Ocares-Riquelme J., Poon P.S., Montaña R., García X., Campos K., Hernández-Garrido J.C., Titirici M.M. (2019). C-doped anatase TiO_2_: Adsorption kinetics and photocatalytic degradation of methylene blue and phenol, and correlations with DFT estimations. J. Colloid Interface Sci..

[34] Martindale B.C.M., Joliat E., Bachmann C., Alberto R., Reisner E. (2016). Clean donor oxidation enhances the H_2_ evolution activity of a carbon quantum dot-molecular catalyst photosystem. Angew. Chem. Int. Ed..

[35] Loryuenyong V., Buasri A., Srilachai C., Srimuang H. (2012). The synthesis of microporous and mesoporous titania with high specific surface area using sol-gel method and activated carbon templates. Mater. Lett..

[36] Wen Z., Wang X., Mao S., Bo Z., Kim H., Cui S., Lu G., Feng X., Chen J. (2012). Crumpled nitrogen-noped graphene nanosheets with ultrahigh pore volume for high-performance supercapacitor. Adv. Mater..

[37] de Britto D., Campana-Filho S.P. (2007). Kinetics of the thermal degradation of chitosan. Thermochim. Acta.

[38] Li J., Wang J., Liu J., Li Y., Ma H., Yang J., Zhang Q. (2020). Facile synthesis of multi-type carbon doped and modified nano-TiO2 for enhanced visible-light photocatalysis. RSC Adv..

[39] Shen T., Wang Q., Guo Z., Kuang J., Cao W. (2018). Hydrothermal synthesis of carbon quantum dots using different precursors and their combination with TiO_2_ for enhanced photocatalytic activity. Ceram. Int..

[40] Yu X., Liu J., Yu Y., Zuo S., Li B. (2014). Preparation and visible light photocatalytic activity of carbon quantum dots/TiO_2_ nanosheet composites. Carbon.

[41] Martindale B.C.M., Hutton G.A.M., Caputo C.A., Prantl S., Godin R., Durrant J.R., Reisner E. (2017). Enhancing light absorption and charge transfer efficiency in carbon dots through graphitization and core nitrogen doping. Angew. Chem. Int. Ed..

[42] Li D., Cheng X., Yu X., Xing Z. (2015). Preparation and characterization of TiO_2_-based nanosheets for photocatalytic degradation of acetylsalicylic acid: Influence of calcination temperature. Chem. Eng. J..

[43] Yu J., Wang B. (2010). Effect of calcination temperature on morphology and photoelectrochemical properties of anodized titanium dioxide nanotube arrays. Appl. Catal. B Environ..

[44] Acevedo-Peña P., González I. (2013). TiO_2_ photoanodes prepared by cathodic electrophoretic deposition in 2-propanol: Effect of the electric field and deposition time. J. Solid State Electrochem..

[45] Chen W., Jiang L., Pathak R., Wu F. (2019). Sb_2_S_3_/TiO_2_ Heterojunction solar cells based on carbon electrode with higher photocurrent. ECS J. Solid State Sci. Technol..

[46] Mirtchev P., Henderson E.J., Soheilnia N., Yip C.M., Ozin G.A. (2012). Solution phase synthesis of carbon quantum dots as sensitizers for nanocrystalline TiO_2_ solar cells. J. Mater. Chem..

[47] Maiaugree W., Lowpa S., Towannang M., Rutphonsan P., Tangtrakarn A., Pimanpang S., Maiaugree P., Ratchapolthavisin N., Sangaroon W., Jarernboon W. (2015). A dye sensitized solar cell using natural counter electrode and natural dye derived from mangosteen peel waste. Sci. Rep..

[48] Meneghetti M., Talon A., Cattaruzza E., Celotti E., Bellantuono E., Rodríguez-Castellón E., Meneghetti S., Moretti E. (2020). Sustainable organic dyes from winemaking lees for photoelectrochemical dye-sensitized solar cells. Appl. Sci..

[49] Ashok A., Mathew S.E., Shivaram S.B., Shankarappa S.A., Nair S.V., Shanmugam M. (2018). Cost Effective Natural Photo-Sensitizer from Upcycled Jackfruit Rags for Dye Sensitized Solar Cells. J. Sci. Adv. Mater. Devices.

[50] Hosseinnezhad M., Gharanjig K., Ghahari M., Nasiri S., Fathi M. (2024). Investigation of the Use of Food Waste in Renewable Energy Production: Extraction, Fabrication and Characterization of Natural Photosensitizers in DSSCs. Sustain. Energy Technol. Assess..

[51] Xu S., Huang P., Zhong W., Luo Y., Yan Z., Liu L., Xiao Z. (2023). Dual-Biowaste-Based Dye-Sensitized Solar Cells Using Natural Chlorophyll Dye and Natural Counter Electrode Derived from Fallen Leaves. Appl. Phys. A.

[52] El-Deen S.S., Hashem A.M., Abdel Ghany A.E., Indris S., Ehrenberg H., Mauger A., Julien C.M. (2018). Anatase TiO_2_ nanoparticles for lithium-ion batteries. Ionics.

[53] Milenov T., Avramova I. (2015). Deposition of graphene by sublimation of pyrolytic carbon. Opt. Quant. Electron..

[54] Matos J., García A., Poon P.S. (2010). Environmental green chemistry applications of nanoporous carbons. J. Mater. Sci..

[55] Fernández de Cordoba M.C., Matos J., Montaña R., Poon P.S., Lanfredi S., Praxedes F.R., Hernández-Garrido J.C., Calvino J.J., Rodríguez-Aguado E., Rodríguez-Castellón E. (2019). Sunlight photoactivity of rice husks-derived biogenic silica. Catal. Today.

[56] Velasco L.F., Carmona R.J., Matos J., Ania C.O. (2014). Performance of activated carbons in consecutive phenol photooxidation cycles. Carbon.

[57] Ania C.O., Armstrong P.A., Bandosz T.J., Beguin F., Carvalho A.P., Celzard A., Frackowiak E., Gilarranz M.A., László K., Matos J. (2020). Engaging nanoporous carbons in “Beyond Adsorption” applications: Characterization, challenges and performance. Carbon.

[58] Atienzar P., Valencia S., Corma A., Garcia H. (2007). Titanium-containing zeolites and microporous molecular sieves as photovoltaic solar cells. Chemphyschem.

