# Supplementary material for: Agave Bagasse as an Eco-Friendly Template for the Microwave-Assisted Synthesis of C@TiO2 Photoelectrodes
Source: Molecules. 2026 Jul 7;31(13):2399. doi: 10.3390/molecules31132399 (PMC13363023; doi:10.3390/molecules31132399)
Supplement: Supplementary file 1 [file molecules-31-02399-s001.zip › molecules-4335586-supplementary.pdf]

Table S1. CHONS analysis of agave bagasse acid hydrolysate (AHAB)

	Proximate and ultimate analysis				
**PAB**	C (%)	N (%)	H (%)	S (%)	O (%)
	45.7 ± 0.6	2.7 ± 0.2	3.2 ± 0.3	0.27 ± 0.04	48.1 ± 0.3

[a] Oxygen content O (%) was estimated by difference between 100% - C (%) – N (%) – H (H) – S (%)

Table S2. Compositional analysis of agave bagasse acid hydrolysate (AHAB)

**Parameters**	**Value (% dry basis)**
Cellulose	26.30 ± 0.03
Hemicelulloce	32.21 ± 0.01
Liginin	15.01 ± 0.06
Soluble compounds (sucrose + glucose + fructuose)	8.21 ± 0.01
Proteina [a]	12.21 ± 0.01
Ashes [b]	6.06 ± 0.01

[a] Protein was determinate in function to the nitrogen percentage. [b] Incombustible solid material was determinate through gravimetric calcination.

Table S3. Elemental analysis of agave bagasse acid hydrolysate (AHAB) from inductively coupled plasma-optical emission spectroscopy (ICP-OES).

Elemental composition (mg/g dried AHAB)						
Ca	Zn	Fe	Al	Na	P	Si
28.8 ± 0.4	6.6 ± 0.3	5.0 ± 0.3	7.1 ± 0.4	5.6 ± 0.3	40.5 ± 0.4	13.5 ± 0.3

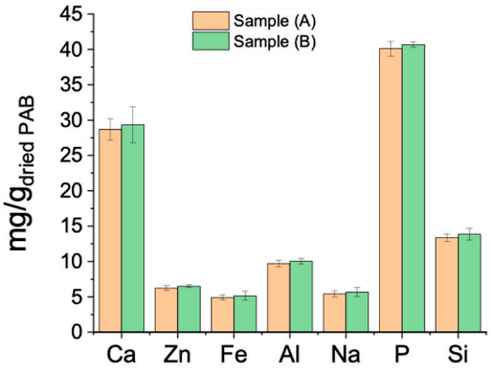

Figure S1. Elemental composition of powders of Agave Bagasse (PAB).

(Eq. S1)

$$i_p = 2.69x10^5 n^{3/2} \text{Apea} C_0 D_{redox}^{1/2} \upsilon^{1/2}$$

Definition of the electrochemical parameters in **Eq. (1):**
i_p: the current intensity of peak
n: electrons transferred in the electrochemical interface
A_{pea}: photoelectroactive area of working electrode
C_o: molar concentration of redox probe, $K_4[Fe(CN)_6]$
D_{redox}: diffusion coefficient of redox probe, $K_4[Fe(CN)_6]$
v: scan rate on electrochemical system

(Eq. S2)

$$E_p = \left(\frac{2.303RT}{n * k_{pct}^0 * F}\right) log v + C$$

Definition of the electrochemical parameters in **Eq. (2):**
E_p: value potential of the peak
R: universal constant of ideal gases
T: temperature
n: electrons transferred in the electrochemical interface
k_{pct}^0: electrochemical kinetic
F: Faraday constant
ν: scan rate on electrochemical system.
C: molar concentration of redox probe, $K_4[Fe(CN)_6]$

The experimental Ep values were obtained from a series of cyclic voltammograms at different sweep speeds and in the presence (ON) or absence (OFF) of UV-Vis light.
